# Perioperative microbiota at the colorectal anastomosis: local host–microbe interactions in healing and leak

**DOI:** 10.3389/fsurg.2026.1895315

**Published:** 2026-08-07

**Authors:** Lu Hou, Yuanyu Wu, Ruizhi Hou, Yafei Li, Zheyu Song

**Affiliations:** 1 School of Basic Medical Sciences, Changchun University of Chinese Medicine, Changchun, China; 2 Department of Gastrointestinal, Colorectal and Anal Surgery, China-Japan Union Hospital of Jilin University, Changchun, China

**Keywords:** anastomotic leak, colorectal anastomosis, colorectal surgery, host-microbe interactions, perioperative microbiota, wound healing

## Abstract

Anastomotic leak remains a major complication after colorectal resection, but established clinical and technical risk factors do not fully explain why some repairs fail during early healing. This review treats perioperative microbiota as part of a local host-microbe wound environment at the colorectal anastomosis rather than as a broad gut microbiome signal. Literature was identified through PubMed and Web of Science searches covering 1 January 2010–6 July 2026, using terms combining colorectal anastomosis, anastomotic leak, microbiota, microbiome, and host-microbe interactions. Evidence was tiered by directness to the anastomotic wound, distinguishing stool-based proxy signals from mucosal, tissue-adjacent, and functional measurements. Stool and broader luminal profiles provide indirect signals for suture-line events unless paired with site-aware sampling. Mucosal, mucus, and tissue-adjacent studies move closer to the repair site but remain mostly exploratory and associative. Animal and *in vitro* work supports plausible mechanisms, including collagenase activity, biofilm-like persistence, metabolite signaling, inflammatory modulation, and extracellular matrix injury, but does not establish human clinical causality. Current evidence justifies better perioperative sampling, function-aware microbial analysis, and integration with conventional leak-risk factors; it does not justify routine microbiome-guided risk stratification or leak-prevention interventions. The most useful next step is longitudinal, site-aware human work anchored to standardized leak outcomes and surgical context.

## Introduction

Anastomotic leak remains among the most consequential complications after colorectal resection. Failure of a colorectal repair can extend beyond a local defect, leading to pelvic sepsis or generalized septic complications, drainage or reoperation, prolonged recovery, stoma-related consequences, delayed oncologic treatment, increased cost, and mortality risk (1–4). For surgeons and patients, leak is not a marginal technical event but a complication that can redirect the postoperative course.

Surgical technique, tissue perfusion, anastomotic level, diversion, nutritional reserve, local disease state, and patient-level risk remain central to anastomotic healing (3, 5). The microbiota question should be placed within that surgical context rather than used to replace it. Perioperative microbiota are most relevant when they are tied to the biology of the colorectal repair itself: the injured mucosal and submucosal wound, the local host response, and the microbial communities or products that may be present near the healing surface.

This review focuses on perioperative microbiota at or near the colorectal anastomosis, local host-microbe interactions, and implications for anastomotic healing and leak risk. The most relevant human samples for this question are site-aware or tissue-adjacent samples, such as anastomotic-tissue mucus, mucosa near the repair, luminal-near-site material, drain fluid, or other tissue-adjacent specimens. Stool and broader gut microbiome profiles remain useful for identifying perioperative associations, but they are lower-directness proxy signals unless paired with local sampling, functional measurements, or host-response readouts close to the anastomotic wound.

## Methods

This article is a focused narrative review rather than a formal systematic review. The purpose was to synthesize a heterogeneous body of clinical, human-associated microbiome, preclinical, and mechanistic literature according to directness to the anastomotic wound and strength of inference, not to estimate a pooled effect size. A formal systematic review was not selected because the central question is conceptual and translational: how indirect stool-based signals, site-aware human data, animal models, *in vitro* mechanisms, and clinical outcome evidence should be interpreted without treating them as equivalent forms of evidence.

Searches were performed in PubMed and Web of Science for publications from 1 January 2010 to 6 July 2026, using combinations of colorectal anastomosis, anastomotic leak, anastomotic healing, microbiota, microbiome, host-microbe, collagenase, biofilm, metabolite, mucosa, mucus, oxygen, hypoxia, ischemia, perfusion, and related terms. Reference lists of key clinical studies, microbiome studies, and relevant systematic reviews were hand searched. Prior systematic reviews were used to orient clinical risk-factor, economic-burden, bowel-preparation, oral-antibiotic, and collagenase-related evidence, but they were not treated as direct proof of local anastomotic host-microbe causality.

Studies were included when they were directly relevant to anastomotic-site biology, perioperative microbiome evidence, host-microbe mechanisms in colorectal healing, or clinical exposures relevant to leak risk. Animal, *in vitro*, and *ex vivo* studies were included only when they addressed anastomotic healing, barrier repair, matrix remodeling, microbial virulence behavior, oxygen/perfusion-related host-microbe biology, or related local mechanisms. These studies were interpreted as mechanistic support and not as human clinical causal evidence. Evidence was tiered by directness to the anastomotic wound environment, distinguishing clinical outcome evidence, stool or broad gut microbiome evidence, mucosal/tissue-adjacent human evidence, human metabolite or function-associated evidence, animal and *in vitro* mechanistic evidence, microbiome methods evidence, and review-level orientation (Table 1).

**Table 1:** Evidence hierarchy and inferential boundaries for host-microbe studies of colorectal anastomotic healing.

Evidence tier	Directness to wound	Can support	Cannot support	Use in this review
Clinical leak outcome studies	Low for microbiome mechanism	Leak burden, severity, conventional risk factors, and exposure-outcome associations	Microbiome mediation or local host-microbe causality	Defines clinical stakes and covariates
Stool or broad gut microbiome studies	Low	Gut-level associations and perioperative proxy signals	Suture-line biology, wound-surface function, or local causality	Generates hypotheses; interpreted cautiously
Mucosal, tissue-adjacent, or site-aware human studies	Moderate to high	Local relevance and candidate wound-associated signals	Established causality, timing of failure, or routine prediction	Closest human evidence
Human metabolite or function-associated studies	Variable	Links between microbial signals, metabolites, inflammation, barrier repair, or matrix remodeling	Direct local suture-line proof when sampled away from the wound	Connects microbiology with host repair pathways
Animal and *in vitro* mechanistic studies	Mechanistic, non-human	Controlled tests of collagenase, MMP-linked injury, ischemia, oxygen/perfusion, diet, or microbial behavior	Human clinical causality or practice-changing prevention	Supports biological plausibility
Microbiome methods evidence	Methodological	Limits of sampling, sequencing, contamination, and predicted functional profiles	Leak mechanism or biological causation	Controls overinterpretation
Review-level sources	Background	Orientation, terminology, and prior synthesis	Primary proof for specific causal claims	Context only

Evidence tiers are separated to avoid treating clinical outcome studies, stool-based microbiome associations, local or tissue-adjacent sampling, preclinical mechanisms, microbiome methods papers, and review-level synthesis as equivalent forms of evidence. Stool and broad gut microbiome findings should be interpreted as indirect unless paired with site-aware sampling or function-aware validation.

### The colorectal anastomosis as a local host–microbe wound environment

A colorectal anastomosis is more than a technical join between two bowel ends. It is a newly injured mucosal and submucosal wound placed beside a dense luminal microbial reservoir. At the suture or staple line, epithelial continuity is interrupted, mucus architecture is disturbed, extracellular matrix is exposed, and tissue perfusion may vary around the repair. Anastomotic healing literature emphasizes inflammatory, proliferative, and remodeling phases, and human colorectal data link reduced rectal-stump microperfusion with leak risk (6, 7). Local inflammation is expected and necessary, but its intensity, duration, and spatial distribution may influence whether repair proceeds toward stable integration or tissue breakdown. Human translational data linking gut microbiota with mucosal proinflammatory cytokines in colorectal cancer surgery support this host-response model, while still falling short of direct causal proof at the suture line (8). The biological question is how microbial communities and microbial products near injured tissue behave while the host attempts to restore barrier integrity and tensile strength.

This local model matters because anastomotic healing depends on events at the wound surface and in the immediately adjacent tissue. Barrier restitution involves epithelial migration, re-epithelialization, and restoration of a controlled host-luminal boundary (6, 9). Inflammatory signaling has to contain contamination and clear damaged tissue; when prolonged or excessive, it may amplify proteolysis, edema, or local ischemic injury. Matrix turnover also has to remain balanced: collagen deposition and remodeling are needed for wound strength, whereas excessive matrix degradation can weaken the repair (6). Adhesion, surface persistence, metabolite production, immune activation, and enzyme activity are therefore relevant only when they can be connected to local repair biology.

The distinction between local peri-anastomotic biology and stool-based or broader perioperative inference is essential. Fecal profiles can describe the broader intestinal ecosystem surrounding an operation, but fecal evidence remains indirect for local anastomotic biology (10). Perioperative rectal-swab or other human-associated microbiome studies may help identify community shifts around surgery, but such data do not, by themselves, establish wound-surface biology at the suture line (11, 12). Site-aware studies using stapled-anastomosis sampling, intraoperative anastomotic-tissue mucus, mucosal swabs from resection margins, or colorectal mucosal samples move closer to the relevant compartment, but their findings remain associative, proof-of-principle, or tissue-adjacent rather than definitive local causality (13–16). Treating stool as a direct readout of the anastomosis would blur the central biological problem.

The perioperative period also makes this wound environment rapidly changing. The operation alters the repair site through mobilization, bowel transection, stapling or suturing, tissue handling, local bleeding, and shifts in perfusion. Mechanical bowel preparation (MBP) and antibiotics can change luminal burden and microbial selection pressure, although clinical outcome evidence should not be assumed to prove a microbiome-mediated mechanism. After reconstruction, the anastomosis is exposed to changing luminal flow, residual contents, blood products, oxygen and nutrient gradients, bile acids, mucus disruption, and host inflammatory signals. These factors interact at the wound rather than acting as isolated exposures. A locally hypoperfused segment, for example, may create a repair environment in which epithelial restitution is delayed, inflammatory signaling persists, and microbial behaviors that would otherwise be contained become more consequential. Experimental ischemic colon-resection data support the concept that microbiota phenotype can interact with impaired perfusion, but this remains animal evidence rather than human clinical causality (17).

**Figure 1:**
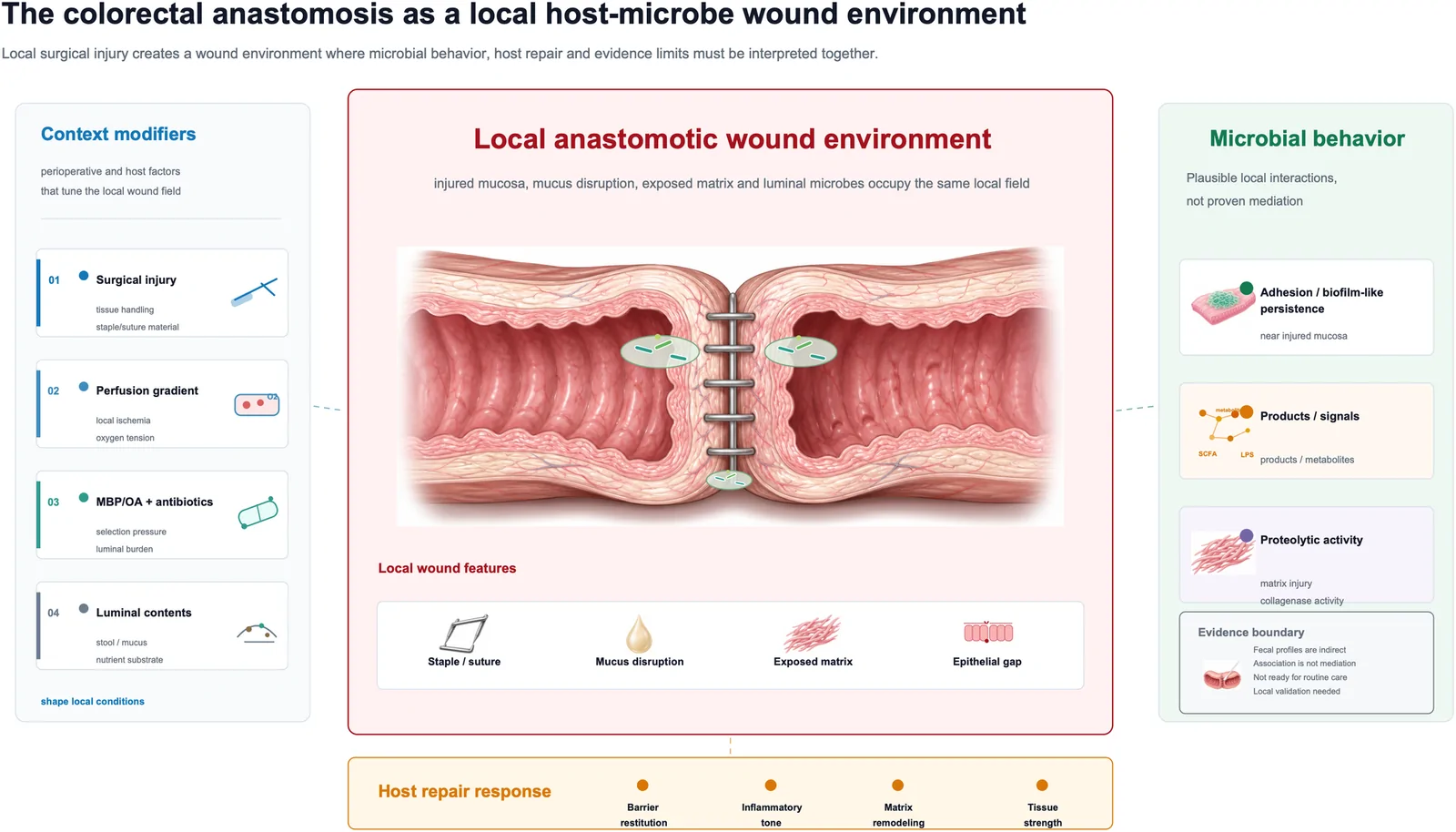
The colorectal anastomosis as a local host-microbe wound environment. Surgical injury, perfusion gradients, mucus disruption, luminal contents, bowel preparation, and antibiotics shape the perioperative wound niche. Microbial behavior may intersect with epithelial restitution, inflammatory tone, matrix remodeling, and tissue strength. Arrows indicate plausible interactions and evidence-directness pathways rather than proven clinical causality.

This local wound model keeps dysbiosis, virulence-related behavior, metabolites, surface persistence, and other candidate mechanisms tied to the repair site rather than to broad luminal imbalance alone. The evidence is not equal across these mechanisms, and direct human anastomotic-site data remain limited, but the central question remains clinically grounded: which microbial and host processes near the colorectal repair may make durable closure more or less likely? The model is summarized in Figure 1.

### Microbial behavior at the anastomotic site

Dysbiosis at a colorectal anastomosis should be understood as a hypothesis about altered wound ecology rather than as a composition-only label. The operation creates a short-lived wound niche: mucus is disrupted, matrix is exposed, blood products and necrotic debris may be present, oxygen tension and nutrient gradients change, and local inflammation reshapes the surface on which microbes encounter host tissue. MBP, oral or systemic antibiotics, bowel transection, luminal flow, and postoperative diet can further change selection pressure. Under these conditions, the useful question is not whether the community differs from baseline, but whether perioperative selection pressure and tissue injury create conditions in which damaging microbial behavior could emerge near the repair.

Compositional signals alone do not explain wound failure. Human preoperative gut, fecal, or broader gut microbiome studies have reported differences between patients with and without anastomotic leakage (10, 18, 19), but such findings remain indirect for local suture-line biology unless the sampling is site-aware. Studies of stapled anastomoses, anastomotic-tissue mucus, mucosal swabs from the anastomotic extremities, or mucosal cancer-associated microbes bring the sampling closer to the wound compartment (13–16). These studies can identify candidates and justify local follow-up; they do not, by themselves, establish function, timing, or causality at the repair.

Surface persistence at injured tissue is a plausible mechanism, but current human colorectal evidence does not establish biofilm-like communities as a decisive cause of leakage. What would strengthen this hypothesis is not additional stool-based taxonomic surveys, but studies that combine local sampling with histological confirmation of surface-adherent communities and direct functional measurements at the suture line.

Virulence-related behavior requires the same restraint. Collagenase-related activity, proteolysis, epithelial injury, and other matrix-damaging functions are plausible mechanisms because early anastomotic strength depends heavily on balanced collagen turnover and controlled inflammation. Review-level synthesis has highlighted collagenase-producing bacteria as a recurring theme in leak-associated contexts (20). Mechanistic work has linked bacterial collagen degradation and MMP9 activation to intestinal anastomotic leak under experimental conditions, and screening studies have identified collagenolytic bacteria isolated from human clinical samples with possible relevance to leak and wound-complication hypotheses (21, 22). Preclinical work has also shown that manipulating bacterial collagenase-related activity can alter anastomotic leak outcomes in defined models (23, 24). Other experimental studies suggest that injured intestinal tissue can select for virulence adaptation in controlled systems and that *Fusobacterium nucleatum* can induce MMP9-linked epithelial responses in preclinical anastomosis models (25–27). Together, these data move the discussion from presence to behavior. They should not be read as proof that one organism, one enzyme, or one pathway is the established cause of leakage in patients.

The strongest interpretation is therefore functional but not deterministic. Perioperative dysbiosis may matter when it changes the local balance at the wound: making pathobiont behavior harder to contain, selecting for organisms that tolerate the postoperative environment, or allowing surface persistence and proteolytic activity close to vulnerable tissue. Antibiotics and bowel preparation fit this argument as selection pressures and clinical exposures, not as proof of a microbiome-mediated mechanism. Likewise, a leak-associated taxon in stool should be treated as a marker or hypothesis unless paired with evidence of local abundance, timing before tissue breakdown, and measured microbial function at or near the anastomosis.

### Microbial metabolites, barrier repair, inflammation, and matrix remodeling

At an injured colorectal anastomosis, microbial products and metabolites should be understood as candidate modifiers of the local repair environment. Direct local human metabolite evidence remains limited, so the discussion below frames these signals as mechanistic hypotheses about barrier recovery, inflammation, and tissue integration—not as established clinical determinants of leak.

Barrier restitution is the most immediate host process in which this candidate biology could matter. Early healing requires epithelial migration across the injured surface, re-epithelialization, and restoration of a controlled boundary between luminal contents and the underlying tissue (6, 9). General epithelial-repair and anastomotic-healing literature supports the host requirement for barrier restitution, but evidence that specific microbial products improve this process at the colorectal suture line remains thin. At present, microbial-product effects on epithelial energy balance, mucus-associated recovery, or epithelial stress should be read as plausible mechanisms to test, including the possibility that local chemical conditions may delay closure of the luminal-tissue interface. The local criterion is whether signals near the repair make early barrier recovery more or less likely.

Inflammatory tone is similarly shaped by local signals rather than by microbial presence alone. Some degree of inflammation is necessary after anastomosis creation because the wound has to contain contamination, clear damaged tissue, and coordinate repair (6). That response becomes harmful when it is excessive, persistent, or spatially poorly controlled, because the same pathways that protect the wound can also amplify epithelial injury, edema, and proteolysis. Microbial products and host responses to those products may modify this balance by influencing epithelial stress programs, innate immune activation, and mucosal immune signaling. The strength of evidence is not uniform across pathways: TLR-mediated and inflammasome-associated signaling have the strongest human translational support in surgical contexts, whereas specific metabolite-receptor interactions such as indole-AhR-IL-22 remain primarily supported by experimental models. Human translational work has linked gut microbiota with mucosal proinflammatory cytokine patterns relevant to anastomotic healing without establishing a single causal pathway (8). Emerging metabolite-function work combines human fecal metabolomics with experimental AhR- or IL-22-associated pathway testing; it supports mechanistic interest in indole-linked biology rather than direct human clinical proof at the anastomotic site (28).

The same caution applies to extracellular matrix remodeling and tissue strength. Durable healing depends on more than epithelial closure: collagen deposition, collagen turnover, fibroblast-related repair activity, and control of excessive matrix degradation all have to remain sufficiently coordinated for the anastomosis to gain strength (6). Reducing the problem to a single collagenase mechanism is less useful than recognizing that wound strength is determined by the overall repair environment. Experimental colonic-anastomosis studies support the importance of MMP activity and proteolytic control in early repair, but they support host matrix biology rather than microbiome causality (29, 30). Microbial products could still matter indirectly by sustaining inflammation, worsening epithelial disruption, shifting local proteolytic balance, or changing the host response in tissue that is already hypoperfused or mechanically stressed. Preclinical *Fusobacterium nucleatum* work provides one example of microbial stimulation of epithelial MMP9-linked injury, but this remains animal and *in vitro* mechanism rather than human clinical causality (27). Human-associated microbiota plus experimental epithelial work provides translational support for a microbiota-collagen-synthesis hypothesis, but not direct clinical causality in patients (31).

These signals arise within a clinical context. Perioperative conditions, including antibiotic exposure, bowel preparation, diet, residual luminal contents, and the physical environment created by surgery, help shape which metabolites and microbial products are present near the repair. Direct assays of microbial products, metabolite-linked host responses, barrier function, or matrix-related readouts generally carry more mechanistic weight than taxonomic shifts or marker-gene predicted functional profiles alone (32). Measurements from stool, blood, or broad luminal compartments remain useful context, but they are proxy evidence for wound-surface events unless connected to site-aware sampling or local host-response measurements.

### Clinical factors that shape the perioperative microbial environment

Perioperative factors are relevant to this review only when they shape the local microbial and chemical conditions at the anastomotic wound. The repair is exposed to changing luminal burden, antimicrobial pressure, nutrient availability, contamination, inflammatory signaling, and tissue vulnerability over a short postoperative interval in which barrier restitution and matrix stabilization are still incomplete. Not every exposure acts through the microbiome; the relevant question is how antibiotics, bowel preparation, nutrition and host reserve, diversion, neoadjuvant therapy, perfusion, and operative handling modify or confound the wound environment in which microbial behavior is interpreted.

MBP and oral antibiotics are clear perioperative selection pressures. They can alter luminal biomass, change which organisms are suppressed or favored, and modify microbial signals reaching an injured mucosal surface during early healing. Perioperative rectal cancer data and colonic mucosa-associated microbiome data show that these exposures can shift rectal or mucosal communities (11, 33). Clinical outcome studies and meta-analyses evaluate bowel-preparation or oral-antibiotic strategies against SSI, morbidity, and leak-relevant endpoints, but such results should not be read as proof of microbiome-mediated leak prevention (34–40).

Systemic antibiotics belong in the same bounded category. Their primary purpose is prophylaxis and infection control, not leak-specific microbiome engineering. Randomized prophylaxis data comparing systemic with oral-plus-systemic antibiotic strategies should therefore be read as clinical outcome evidence rather than proof of local microbiome engineering (41). Antimicrobial exposure helps define the wound environment in which microbial associations are observed, but its local effect on healing biology remains difficult to predict.

Diet, nutrition, frailty, and metabolic reserve matter when they change wound context. Experimental exposure models support the general point that diet and bowel preparation can alter colon microbiota and anastomotic healing, although this remains animal evidence rather than direct human proof (42, 43). Poor nutrition is recognized in colorectal leak risk-factor literature, and colorectal cancer surgery data link sarcopenia, frailty, and nutritional depletion with adverse postoperative outcomes (3, 44). These factors should be interpreted as wound-context modifiers and confounders of microbial signals, not as established microbiome mechanisms.

Local disease state and operative context also modify the biology into which the anastomosis is placed. Obstruction, active inflammation, contamination burden, uneven tissue perfusion, tissue handling, anastomotic construction, diversion, and neoadjuvant therapy can all change local exposure and vulnerability (3, 5, 7). These conditions may shift which organisms reach the repair, which products accumulate there, and how the host responds. A perioperative microbial signature may therefore reflect a modifiable exposure, a marker of tissue stress, or both.

### Translational implications for risk stratification and leak prevention

Translational interest is understandable because anastomotic leak remains clinically important in colorectal surgery (4). Existing risk assessment is still imperfect: technical factors, perfusion, anastomotic level, diversion, nutritional status, and local disease context remain central to surgical judgment, yet the conventional risk-factor literature does not fully resolve why some individual repairs fail (3, 5). Microbiology is best considered a candidate adjunct to that framework, not an established predictive layer, because it may capture aspects of the local wound environment that routine variables measure only indirectly.

Within that frame, microbial, host-microbe, or metabolite-associated signals function as candidate risk markers. Human-associated studies have reported perioperative or baseline microbial differences, including intraoperative anastomotic-tissue mucus, mucosal microbiome proof-of-principle, and gut microbiota prediction approaches; current signals remain exploratory and often indirect for the anastomotic site itself (10, 11, 14, 45, 46). The clinically relevant question is not whether microbiology can support a standalone prediction claim, but whether a microbial or host-microbe measure adds interpretable information beyond the factors surgeons already use to judge leak risk.

Current human evidence remains too indirect and exploratory to support routine microbiological risk stratification at the colorectal anastomosis (10, 11). Before any bedside use, proposed risk models would need prospective cohorts with adequate sample sizes, standardized leak definitions, site-aware sampling at clinically relevant time points, external validation, demonstration of added value beyond conventional clinical variables, feasibility assessment, and evidence of clinical utility including decision-curve evaluation. Until these criteria are met, microbiology is better viewed as a source of hypotheses and interpretable clinical context than as a basis for practice change.

Preventive implications should be handled with the same restraint. Microbiology may help refine trial design, identify biologically coherent subgroups, or suggest when a preventive strategy deserves focused testing. Clinical outcome evidence indicates that some perioperative practices can reduce leak rates in selected analyses or have been tested in rectal-resection trials, but that should not be read as proof of microbiome-mediated prevention (38–40). At present, the human microbiome literature is more useful for hypothesis generation and trial design than for routine implementation (10, 11), and routine microbiome-guided prevention is not ready for standard colorectal care.

### Limitations of current evidence and key research gaps

The evidence linking perioperative microbiota to colorectal anastomotic healing is biologically plausible but methodologically uneven. Clinical outcome studies define leak burden and conventional risk factors, but they rarely measure the microbial wound environment directly. Human microbiome studies provide association-level evidence and are often limited by small cohorts, heterogeneous sampling, and incomplete functional validation. Animal and *in vitro* studies offer stronger mechanistic control, but their relevance depends on how closely the model captures operative anatomy, antibiotics, perfusion, local disease state, and clinical leak definitions.

The main compartment problem is that the colorectal anastomosis is a local wound, whereas much available microbiome evidence comes from stool or broad luminal sampling. Stool profiles can be informative about the intestinal ecosystem surrounding surgery, but they are not direct measurements of the suture line, tissue-adjacent surface communities, mucosal injury, or early matrix remodeling (10). Mucosal, anastomotic-tissue, and resection-margin swab studies move closer to the target compartment, but they remain associative or proof-of-principle rather than definitive local causal evidence (13–16).

Timing is another major source of uncertainty. A microbial profile measured before surgery may reflect baseline host factors, diet, tumor, obstruction, bowel preparation, or antibiotic exposure rather than the wound conditions that develop after anastomosis creation. A sample collected after clinical leak becomes apparent may reflect tissue breakdown, contamination, drainage, antibiotics, reoperation, or systemic inflammation rather than the initiating biology. Longitudinal sampling across the preoperative period, operation, early repair interval, and pre-leak window is therefore essential.

Clinical heterogeneity further constrains interpretation. Anastomotic leak may be defined clinically, radiologically, operatively, biochemically, or by severity grade, and these categories are not interchangeable (1, 2). Colon and rectal procedures, diverted and non-diverted anastomoses, elective and emergency surgery, cancer and inflammatory disease, and neoadjuvant therapy create different wound environments. Study size should also be tied to the intended endpoint. Small site-aware cohorts can justify further sampling, but prediction or mediation claims need prospective, event-driven sample-size calculations based on expected leak incidence, sampling compartment, candidate microbial and host variables, and the expected number of leak events.

Technical differences in microbiome measurement also matter. Sequencing platform, DNA extraction, contamination control, sequencing depth, taxonomic resolution, and bioinformatic pipelines can all affect the organisms reported (47, 48). Predicted functional profiles based on marker-gene data are useful for generating hypotheses, but they are not equivalent to direct evidence of collagenase activity, biofilm formation, metabolite production, epithelial injury, or matrix degradation at the anastomosis (32).

Causality is difficult to establish because temporal ambiguity, confounding, reverse causation, and proxy sampling often coexist. Patients at higher risk of leak may differ in tumor location, neoadjuvant therapy, obstruction, nutrition, antibiotic exposure, bowel preparation, operative complexity, perfusion, diversion, and postoperative care. Stronger causal inference would require evidence that microbial changes occur before breakdown, are linked to measured local function, and add explanatory value beyond established clinical and technical factors.

The most informative human studies would be longitudinal, site-aware, function-aware, and clinically interpretable. They would connect operative details and established risk factors with samples from compartments close to the anastomotic wound at biologically meaningful time points, measure microbial function and host response rather than taxonomy alone, and relate these data to standardized leak outcomes. Until then, perioperative microbiota should be discussed as a plausible contributor to local anastomotic wound biology and a rationale for better translational study design, not as an established clinical determinant of leak or a basis for routine microbiome-guided prevention.

## Discussion

Technical execution, tissue perfusion, anastomotic level, diversion, nutritional reserve, and patient condition remain the foundation of colorectal anastomotic healing and leak prevention. The argument advanced here does not displace that surgical foundation. It adds a local biological layer: the colorectal anastomosis is a healing wound placed beside a dense microbial reservoir, and microbial behavior may become relevant when it intersects with epithelial restitution, inflammatory tone, matrix remodeling, and tissue strength at the repair surface.

The main interpretive value of a local host-microbe model is evidence directness. Stool and broader gut microbiome profiles can identify perioperative associations and generate hypotheses, but they do not directly show what is happening at the suture line. Mucosal, mucus, drain-fluid, tissue-adjacent, and function-aware studies move closer to the biological compartment of interest, although most remain small, exploratory, or associative. Mechanistic animal and *in vitro* studies can test collagenase activity, MMP-linked injury, microbial adaptation, metabolite signaling, and barrier pathways under controlled conditions, but they should not be treated as human clinical proof. Separating these evidence tiers prevents biologically plausible mechanisms from being overstated as established determinants of leak.

Local oxygen biology is one plausible bridge between host physiology and microbial behavior at the anastomosis. Tissue handling, edema, inflammation, vascular disruption, and segmental hypoperfusion can create spatial gradients in oxygen tension and redox state across the early wound surface. These gradients may influence epithelial restitution and matrix remodeling on the host side while also altering the growth and functional behavior of facultative anaerobes and anaerobic microbial communities. This mechanism is attractive because it links an established surgical concern, perfusion, with microbial adaptation at injured tissue. However, direct human evidence connecting peri-anastomotic oxygen gradients, microbial function, and subsequent leak remains limited, so oxygen-dependent microbial adaptation should be presented as a testable mechanism rather than an established clinical pathway.

The translational implication is therefore cautious. Current evidence supports better study design and biologically informed hypothesis generation, not routine microbiome-guided risk stratification or leak-prevention intervention. A clinically useful microbial or host-microbe marker would need to add information beyond conventional variables such as anastomotic level, perfusion, obstruction, neoadjuvant therapy, nutrition, antibiotic exposure, bowel preparation, diversion, operative complexity, and postoperative care. It would also need prospective validation, standardized leak definitions, external testing, and evidence of clinical utility.

The most useful next studies would be longitudinal, site-aware, function-aware, and clinically interpretable. They should connect operative details and established risk factors with samples collected from compartments close to the anastomotic wound at biologically meaningful time points. They should measure microbial function and host response rather than taxonomy alone, and they should relate these data to standardized leak outcomes. Until such evidence is available, perioperative microbiota should be discussed as a plausible contributor to local anastomotic wound biology and a rationale for better translational studies, not as an established basis for routine clinical decision-making.
